# Trends in general practitioner consultations for hand foot and mouth disease in England between 2017 and 2022

**DOI:** 10.1017/S095026882400181X

**Published:** 2025-01-13

**Authors:** Natalia G. Bednarska, Sue Smith, Megan Bardsley, Paul Loveridge, Rachel Byford, William H Elson, Helen E. Hughes, Simon de Lusignan, Daniel Todkill, Alex J. Elliot

**Affiliations:** 1 Real-time Syndromic Surveillance Team, Field Services, UK Health Security Agency, Birmingham, UK; 2 Nuffield Department of Primary Care Health Sciences, University of Oxford, Oxford UK; 3 Royal College of General Practitioners (RCGP) Research and Surveillance Centre (RSC), RCGP, London, UK

**Keywords:** coxsackievirus, enterovirus, hand, foot and mouth disease, general practitioner, syndromic surveillance

## Abstract

Hand, foot and mouth disease (HFMD) is a contagious communicable disease, with a high incidence in children aged under 10 years. It is a mainly self-limiting disease but can also cause serious neurological or cardiopulmonary complications in some cases, which can lead to death. Little is known about the burden of HMFD on primary care health care services in the UK. The aim of this work was to describe trends in general practitioner (GP) consultations for HFMD in England from January 2017 to December 2022 using a syndromic surveillance network of GPs. Daily GP consultations for HFMD in England were extracted from 1 January 2017 to 31 December 2022. Mean weekly consultation rates per 100,000 population and 95% confidence intervals (CI) were calculated. Consultation rates and rate ratios (RR) were calculated by age group and sex. During the study period, the mean weekly consultation rate for HFMD (per 100,000 registered GP patients) was 1.53 (range of 0.27 to 2.47). In England, children aged 1–4 years old accounted for the largest affected population followed by children <1 years old. We observed a seasonal pattern of HFMD incidence during the non-COVID years, with a seasonal peak of mean weekly rates between months of September and December. HFMD is typically diagnosed clinically rather than through laboratory sampling. Therefore, the ability to look at the daily HFMD consultation rates provides an excellent epidemiological overview on disease trends. The use of a novel GP-in-hours surveillance system allowed a unique epidemiological insight into the recent trends of general practitioner consultations for HFMD. We demonstrate a male predominance of cases, the impact of the non-pharmaceutical interventions during the COVID-19 pandemic, and a change in the week in which the peak number of cases happens post-pandemic.

## Introduction

Hand, foot and mouth disease (HFMD) is a contagious communicable disease, with most cases diagnosed in children aged under 10 years [1]. HFMD was first diagnosed in Toronto, Canada, in 1957, but subsequent work recognised the global endemic nature of HFMD. The etiological agents responsible for HFMD belong to the non-polio enterovirus family, including enteroviruses (EV) and coxsackieviruses (CV) [1]. EVs are classified genetically into four categories (A–D) and CV into two groups CV-A and CV-B. Historically, the most common global causes of HFMD were EV-A71 and CVA16, however, currently a higher proportion of HFMD outbreaks are caused by other EVs such as CVA6 and CVA10 [2].

Clinical diagnosis of HFMD is typically based on an assessment of the early presenting symptoms of HFMD, including fever, malaise, loss of appetite, cough, and abdominal pain. Early symptoms are followed by ulcerative lesions of the oral cavity within 1–2 days and classically the presentation of macules and papules of hands and feet appearing later [3]. Atypical manifestations of HFMD (and EV infections in general) can present, particularly in adults, which include future sites for skin manifestations (including the scalp, buttocks and genitalia) and persisting non-dermatological symptoms such as sore throat, fever and asthenia [4, 5]. The incubation period of HFMD is dependent on the serotype of the causative pathogen as well as the age group of the patient, but multiple studies estimated the incubation period to be around 4–8 days, allowing asymptomatic spread of disease in the community [6]. The spread of HFMD can be person-to-person via the faecal-oral route, airborne via infected droplets spread through sneezing or coughing or through contaminated fomites [2]. General infection control measures include frequent handwashing and avoiding close contact with infected individuals, which might be difficult to implement in certain settings such as nurseries.

There is no specific treatment for HFMD other than pain relief management [1]. The disease is usually self-limiting, however, in a small proportion of cases the disease can lead to neurological complications, respiratory failure and in some cases death [7]. Globally, countries in the Asia-Pacific region deal with the highest burden of HFMD, where it is estimated to cause 96,900 (95% CI 40,600 to 259,000) age-weighted disability-adjusted life years per annum [8].

Different causative agents of HFMD can result in more severe outcomes; enterovirus EV-A71 has been known for its virulence, with more severe symptoms including meningitis, encephalitis, and pneumonia [3]. While HFMD is in general a self-limiting mild disease, there is a higher risk of more severe disease occurring in infants younger than 6 months and immunocompromised individuals [9]. In a small proportion of cases, fatal neurological or cardiopulmonary complications can occur. There have also been reports of post-infection neurological sequelae in patients who have recovered from severe infection [10].

In England, there is limited epidemiological information about the community burden of HFMD as the disease is not required to be reported by law. Previous epidemiological studies have shown that HFMD seasonality in England occurs in late summer to early autumn, either sporadically or in regular outbreaks [11]. Meteorological parameters have shown a significant association with the incidence of HFMD in subtropical regions, including mean temperature, rainfall, and relative humidity [12, 13]. Modelling results by a South Korean research group illustrated a direct correlation between the HFMD incidence rate with average temperature and relative humidity [14], therefore the disease potentially has more public health relevance in the context of global warming.

Most HFMD outbreaks happen in childcare centres, nurseries, or within the family setting since HFMD affects mostly children younger than 10 years of age. CVA16 was the main pathogen of HFMD outbreaks in England in 1959 and 1994 [11, 15]. Here, we use routinely available general practitioner (GP) HFMD consultation data to provide an updated epidemiological summary of the HFMD burden on GP practices in England.

## Methods

### Study design and population

This was a retrospective, observational, descriptive analysis of GP consultations for HFMD across England. GP consultation data were sourced from the UK Health Security Agency (UKHSA) GP in-hours syndromic surveillance system. The GP in-hours system collates and monitors GP consultation data for a range of health conditions and diseases as part of the routine UKHSA real-time syndromic surveillance programme [16]. The GP in-hours system uses data from two separate sources [17]. Here, GP in-hours consultations were used from the Royal College of General Practitioners (RCGP) Research and Surveillance Centre (RSC), one of the world’s oldest sentinel networks. RSC data are held on the Oxford- Clinical Informatics Digital Hub (ORCHID) [18]. The study population was all persons who presented to general practices of the RSC [16] between 1 January 2017 to 31 December 2022 inclusive. The mean number of general practices across the period of the study was1,245 practices across England covering a patient population of 11 million [19]. The study dataset included information on primary care demographics such as age and sex.

### Case definition

A case of HFMD was defined as a general practice consultation episode where the GP assigned a Systematised Nomenclature of Medicine Clinical Terms (SNOMED-CT; the terminology system currently used in UK general practice [20]) clinical code inferring a diagnosis of HFMD. The SNOMED-CT clinical codes included for HFMD were as follows: 67171006 Enteroviral vesicular stomatitis with exanthem; 154357002 Hand foot and mouth disease; 175497008 Hand, foot and mouth disease; 186664000 (Hand, foot & mouth disease) or (vesicular stomatitis with exanthem); and 266108008 Enteroviral vesicular stomatitis with exanthem.

### Statistical analysis

Daily counts of GP consultations for HFMD and the GP registered practice population were extracted for each day during the study period by age group and sex from 1 January 2017 to 31 December 2022 inclusive.

The weekly HFMD consultation rate (and 95% confidence intervals; CI) per 100,000 population across England was calculated using the count of HFMD consultations per International Standards Organisation (ISO) week as the numerator and the weekly GP registered population as the denominator. Bank holidays and weekends were removed from the analysis as routine in-hours GP services are largely restricted on these days. The annual HFMD rates were calculated using the annual total count as the numerator and the annual mean population as the denominator.

Time series graphs were used to visualise trends and seasonality of the weekly national consultation rates for HFMD, overall and stratified by age group and sex. Incidence was defined as the total number of HFMD cases divided by the average population size during the study period.

## Results

### Demographic and temporal characteristics

The cumulative sum of HFMD consultations reported through the GP in-hours system in England from 1 January 2017 to 31 December 2022 was 76,386, translating to a mean weekly rate across the whole study period of 2.15 HFMD consultations per 100,000 registered population.

**Figure 1.**
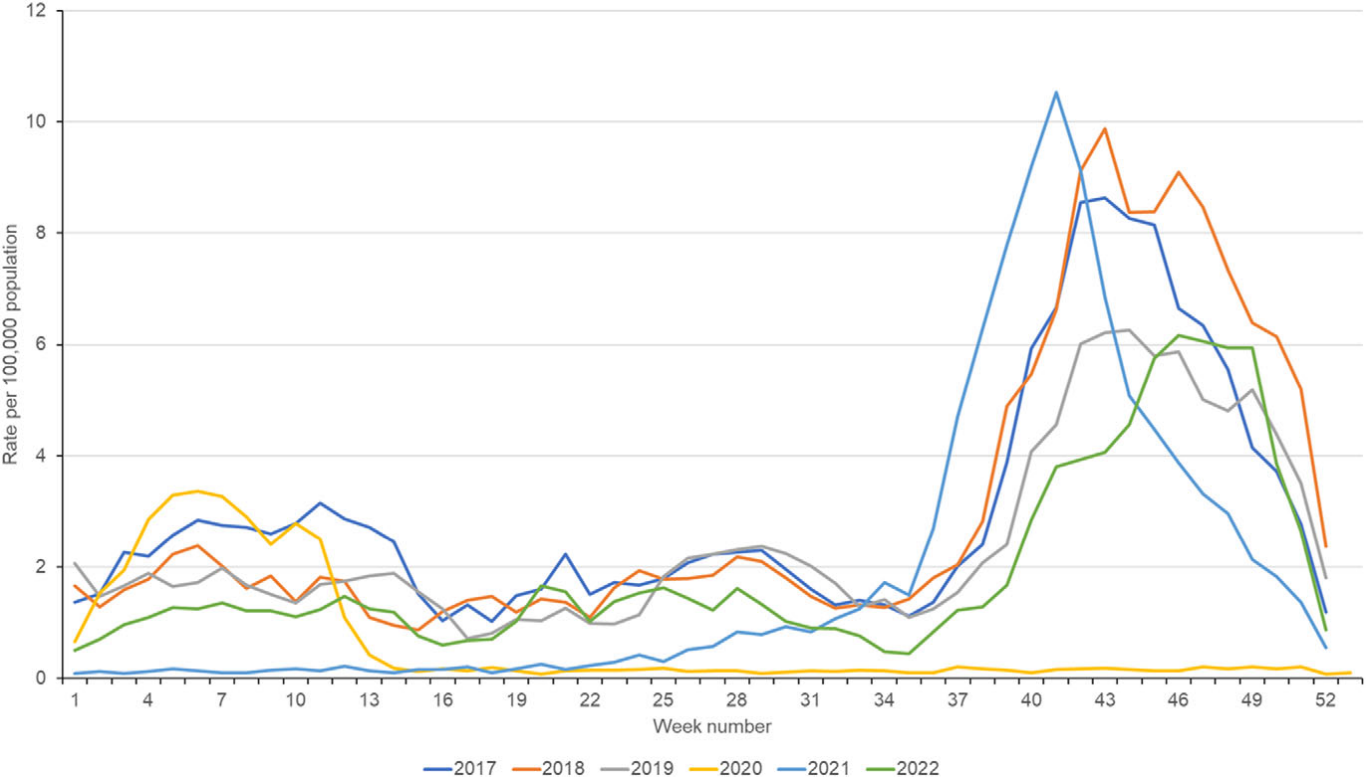
Weekly incidence rate of hand, foot and mouth disease (HFMD) per 100,000 population (all ages), England 2017–2022.

Distinct seasonal HFMD activity was observed across the study period (Figure 1). During the pre-COVID-19 pandemic years (2017–2019), peak HFMD activity (all ages) occurred at weeks 43 and 44, with seasonal activity increasing from baseline activity at weeks 35–36 until peaking approximately 8 weeks later. Peak seasonal activity also varied across the pre-pandemic years; 2017 and 2018 peaked at 8.6 and 9.9 consultations per 100,000 while 2019 had a lower peak at 6.3 per 100,000 (Table 1).

**Table 1.** Hand, foot and mouth disease (HFMD) seasonal activity range

Year	Mean weekly rate^a^	Standard deviation of the mean	Lower 95% CI	Upper 95% CI	minimum rate^a^	maximum rate^a^ (during peak week)	Peak week number
2017	2.47	2.09	1.9	3.03	1.02	8.64	43
2018	2.32	2.6	1.62	3.03	0.86	9.88	43
2019	2.03	1.59	1.61	2.46	0.72	6.26	44
2020^b^	0.27	1.01	0	0.54	0.08	3.36	7
2021^b^	0.62	2.69	−0.1	1.35	0.09	10.53	41
2022^b^	1.47	0.59	1.3	1.62	0.44	6.17	46

a Rate of HFMD consultations per 100,000 registered patients.

b COVID-19 pandemic and post-pandemic years.

During the COVID-19 pandemic years (2020 and 2021), the HFMD consultation rate dropped immediately after the first announcement of COVID-19 restrictions in week 11 (early March) 2020. During 2020 there was no obvious typical epidemic activity or peak observed. The very low HFMD weekly rate continued through 2021 until approximately week 26 when the activity started to increase and then increased sharply from week 35, peaking higher (10.5 consultations per 100,000) and earlier (week 41) than other study years (Figure 1).

During the post-pandemic year (2022), HFMD seasonal activity resumed the expected trend, with mean weekly rates higher than those in 2020 and 2021, but lower than in pre-pandemic years (Table 1). During 2022, the seasonal peak of HFMD activity started later and peaked lower than previous years. The timing of the 2022 seasonal peak was also later than other study years; 2022 peak activity occurred at week 46, however, activity remained high until week 49 when it then decreased, following expected seasonal trends.

When stratified by age, the highest rates of consultations for HFMD in England were observed in children aged 1 to 4 years, followed by infants younger than 1 year old (Table 2). During the whole study period, we registered a total of 76,386 HFMD GP consultations. The average annual consultation rate for the whole study period was 112.1 per 100,000 registered population. Annually HFMD consultation rates in children aged 1 to 4 and < 1 year were the highest in the year 2018 (with a peak of 2,411.5 and 1,799.9 per 100,000, respectively). Activity in age groups 5 years and older was much lower with insignificant activity in adults aged 45 years and over (Table 2).

**Table 2.** Epidemiological characteristics of GP consultations for hand, foot and mouth disease (HFMD) in England, 2017–2022, presented as the annual number of HFMD GP consultations (annual incidence rate per 100,000).

	2017	2018	2019	2020^a^	2021^a^	2022^a^	Mean
Total cases^b^	17,054	17,681	14,247	4,151	11,259	11,994	12,731
Annual incidence	155.4	159.5	126.0	34.8	96.9	99.8	112.1
Age (incidence)							
<1 year	1,852 (1,668.5)	1,973 (1,799.9)	1,494 (1,376.5)	512 (485.8)	1,114 (1,075.9)	1,248 (1,168.2)	1,365.5
1–4 years	12,020 (2,286.6)	12,642 (2,411.5)	10,168 (1,937.5)	2,790 (536.8)	8,424 (1,655.7)	8,693 (1,731.4)	9,122.8
5–14 years	1,679 (128.3)	1,671 (124.1)	1,447 (105.3)	484 (34.7)	823 (58.3)	1,356 (95.3)	1,243.3
15–44 years	1,272 (28.7)	1,189 (26.2)	992 (21.3)	306 (6.4)	799 (16.4)	628 (12.6)	864.3
45–64 years	179 (6.3)	163 (5.6)	123 (4.2)	48 (1.6)	80 (2.6)	54 (1.8)	107.8
>65 years	52 (2.7)	43 (2.2)	23 (1.2)	11 (0.5)	19 (0.9)	15 (0.7)	27.2
Sex (incidence)							
Male (incidence)	9,250 (158.8)	9,609 (164.9)	7,744 (132.9)	2,168 (37.2)	6,278 (107.7)	6,572 (112.8)	6,936.8
Female (incidence)	7,803 (134)	8,072 (139.5)	6,503 (112.4)	1,983 (34.2)	4,980 (86)	5,422 (93.7)	5,793.8
Male to female ratio	1.2	1.2	1.2	1.1	1.3	1.2	1.2

a Total cases include records with unknown age.

b COVID-19 pandemic and post-pandemic years.

**Figure 2.**
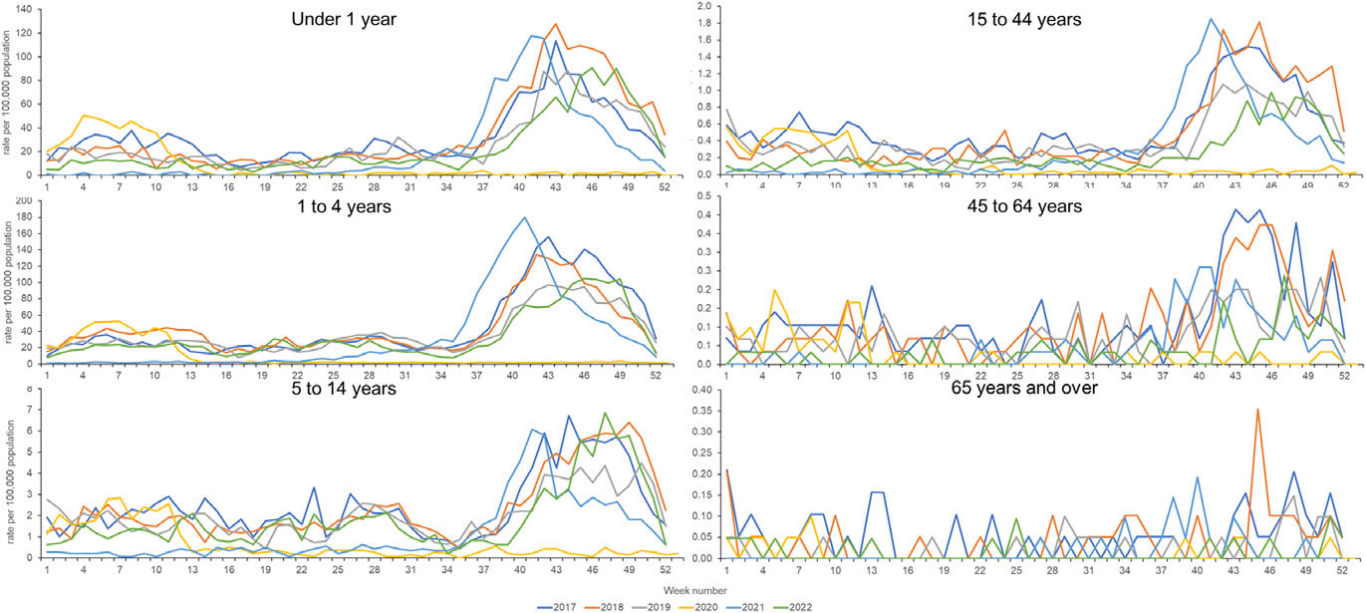
Weekly GP consultation rate of hand, foot and mouth disease (HFMD) per 100,000 population by age group in England (2017–2022).

Temporally, seasonal trends in HFMD incidence across individual age groups generally followed national ‘all ages’ trends (Figures 1 and 2). For children aged <1 year, rates observed during 2018 were higher than in other years, however, for the 1–4 years age group, the highest rates were observed in 2021. Also of note, during 2021 the seasonal peak of HFMD occurred earlier in the 1–4 years age group (compared to <1 year) but was seen to peak later during 2022 (Figure 2). There was also evidence of a possible lag between younger and older age groups, with HFMD consultation activity in adults aged 45 years and over appearing to start and finish later than activity in younger children by a few weeks. However, the small number of HFMD consultations reported in older adults made comparisons challenging.

When stratified by gender, the HFMD consultation rate for males was higher than that seen in females, with the rate ratio consistently illustrating male rates were 20% higher than females across each year of the study period (Table 2).

## Discussion

This study provides an update on recent epidemiological trends in general practice consultations for HFMD in England. Here, we use a national general practice syndromic surveillance system that is routinely used to report on all hazards including infectious diseases (e.g. influenza, COVID-19), environmental impacts (e.g. heatwaves), mass gatherings (e.g. large sporting events) and chemical incidents (e.g. large industrial fires). Laboratory testing and confirmation of HFMD in England is rare and therefore these GP consultation data are a useful proxy for HFMD incidence and can contribute to surveillance of the disease in England. Our study thereby provides valuable insight into the epidemiology and burden of this disease. The data presented here show that HFMD peak week occurred during week 43 or 44 pre-pandemic, During the years 2017–2019 mean weekly rates ranged from 2.03 to 2.47 per 100,000 registered patients. Our data clearly shows the seasonality of HFMD infections for children between 1 and 14 years of age, with the highest incidence during the months of September to January, coinciding with the return of schools following the summer holiday.

Our study shows that the burden of HFMD mainly occurred in children under 5 years of age, which is supported by existing evidence [2, 21–23]. We also demonstrated higher consultation rates in males versus females (average rate ratio of 1.2 during the studied period of time), however, our data support global epidemiological reports where similar findings were made [24, 25]. A research study on the transmissibility of HFMD viruses showed higher indices for male transmissibility and infection rates [26]. The explanation suggested elsewhere in the literature for this phenomenon was the fact that male children are generally more active and exposed to the environment than females [27]. The predominance of disease in younger males is also reported for other conditions, such as asthma, where it has been shown that asthma incidence, prevalence and hospitalisation rates are higher in pre-pubertal boys than girls of the same age, but this trend reverses during adolescence [28, 29].

The current study period spans the COVID-19 pandemic. The impact of the pandemic on the epidemiology of HFMD is clear from our results. GP consultations for HFMD decreased during the early part of 2020, diverging from the seasonal trend observed in other years. Consultation rates remained at very low (near ‘zero’) levels until week 25 of 2021 when HFMD consultations slowly started to increase and return to expected levels. This observation is consistent with reports of the impact of COVID-19 on the circulation of other infectious diseases. In England, non-pharmaceutical interventions (NPIs) were introduced to control the spread of SARS-CoV-2 in the community [30]. An indirect effect of NPIs was the interruption of the transmission chain of other infectious diseases. During the pandemic, surveillance data for respiratory syncytial virus, influenza and gastrointestinal pathogens illustrated very low and out-of-season activity [31]. Our findings support that infection control measures for HFMD (hand washing, restricting close contact, removing cases from close contact settings such as nurseries) are effective measures as the impact of NPIs clearly demonstrated a significant decrease in HFMD circulation. However, it must also be considered that other confounders might have played a role, including changes in the availability of healthcare services and changes in healthcare-seeking behaviour of the public which were also documented during the pandemic [32–34]. Post-pandemic, HFMD seasonality appeared to change. During 2021, the HFMD epidemic curve was earlier than previous years, by 2–3 weeks. However, the following year (2022) saw later activity, 2–3 weeks later than expected. The first HFMD season post-pandemic (2021) might have seen an earlier surge in cases since the lifting of restrictions resulted in schools returning. The cohort of children in the 1–4 years age group would also contain some children not exposed to HFMD pre-pandemic. This is supported by the finding that the 1–4 years age group had the highest incidence during 2021. Further routine surveillance of HFMD is required over the coming years to establish whether the seasonality of HFMD returns to regular pre-pandemic trends.

There was a significant gap since the last epidemiological description of HFMD activity in England, with the last publication dated 1996 [11]. Hereby, we have provided the first update on HFMD epidemiology for 25 years, presenting trends over the recent years 2017–2022. The original 1996 study by Bendig and Fleming utilised a small sentinel network of GPs, the RCGP Weekly Returns Service [11]. This network consisted of 92 sentinel GP practices covering a mean sample population of 614,303 patients. The GP syndromic surveillance system used in our current study involves the same RCGP surveillance network, however, the size of the network and patient population has increased significantly to 1,160 practices, with a mean sample registered patient population of 11 million in 2022 [19], thereby providing a much greater and more representative sample of the population [35].

The peak of HFMD activity described in the 1996 study occurred during ISO week 49, with a peak incidence rate reported at 12.6 consultations per 100,000 population. We present HFMD activity peaking between weeks 41–46 with the maximum mean weekly rate of 10.52 in the year 2021. The suggestion of a lag in HFMD activity between the youngest and oldest age groups is supported by transmission studies of other communicable diseases between these age groups, particularly acute respiratory infections including those caused by respiratory syncytial virus (RSV) [36, 37]. This merits further research into HFMD transmission as there are potentially important implications for infection control advice for older adults who are more at risk of developing severe disease and complications from HFMD, particularly if they are living with or having close contact with younger children who are in the nursery or school setting and therefore more likely to have exposure to the viruses causing HFMD.

Globally the burden and severity of HFMD differs, with particular impact seen in South-East Asian countries, however, in comparison there is a lower burden and relative severity of HFMD in England. Particular strains of enterovirus and coxsackievirus display different neurotropic and deadly propensities worldwide and therefore the surveillance of virus and clinical presentation is of vital importance. In Singapore, HFMD was listed as the top 5 most contagious febrile viral illness amongst children below the age of 5 years [38]. Some of the clinical manifestations of HFMD viruses include more severe aseptic meningitis, encephalitis, acute flaccid paralysis and flaccid myelitis [39]. Coxsackievirus serotype A6 (CVA6) has been identified as a causative agent of the autumn 2008 epidemic outbreak of HFMD in Finland, with the atypical symptom of onychomadesis as a hallmark of this outbreak [40]. HFMD outbreaks are very common in East Asian countries, where now specific reporting systems are implemented as a control measure. Coxsackievirus CV-A16 continues to evolve into more diverse branches as per epidemiological information provided by a Chinese reporting system [41].

In Malaysia, a 1997 outbreak of HFMD resulted in several deaths, after which the country introduced its first control policies including mandatory notification of clusters of cases. Preventative measures inclusive of routine checks of temperature, soles of feet and mouth before allowing children to enter nurseries are also part of the anti-HFMD practice in some countries [3]. In the United States of America, it has been shown that individual serotypes have different temporal patterns of circulation and often are associated with different clinical manifestations [42]. Moreover, the changes in circulating serotypes might be accompanied by large-scale outbreaks, therefore monitoring HFMD occurrence is of high importance.

The serotype EV-A71 was associated with the most infections in Europe, East and South-East Asia. Both coxsackievirus types A16 (CV-A16) and A6 (CV-A6) are found to be prevalent in the USA, Europe [40] and Asia-Pacific with a high pandemic potential [43]. According to historical data, only some sporadic outbreaks were recorded elsewhere to be associated with CV-A10 [44, 45].

A key strength of our study is that we have utilised one of the only routinely available sources of HMFD clinical data. The GP surveillance network is large and covers approximately 18% of the England population. The network has been shown to be representative of the England population thereby ensuring that we have a good cross-section of the population [19]. This system is routinely used for real-time all-hazard surveillance in England and therefore the clinical diagnosis codes used in this study to identify HFMD consultations can be directly applied prospectively for real-time surveillance of HFMD. However, HFMD cases reported here are likely to be an underestimate of total cases in the community. It is likely that mild or asymptomatic HFMD cases will not be reported to primary care or may present to other areas of the National Health Service (NHS) in England. Furthermore, before the development of a classic HFMD vesicular rash, the disease can present with relatively generic symptoms in the early stages of infection meaning that a clinical diagnosis made by a GP might not initially indicate HFMD as the causative diagnosis furthering this underestimate. Finally, a range of other pathogens can be responsible for causing typical and atypical manifestations that might cause a differential diagnosis of HFMD [4].

Increased public awareness of HFMD and emphasising preventative measures such as basic hygiene remain the best means for preventing and controlling cases and outbreaks of HFMD. In England, NHS and local health protection services advise health professionals encountering cases of HFMD to provide advice to patients and their carers, but that no further specialised health protection advice is required [46]. As the majority of cases are not sampled, nor is this a notifiable disease, syndromic surveillance provides a useful tool for measuring the healthcare burden associated with HFMD.

In conclusion, we have described trends in GP consultations for HFMD in England from January 2017 to December 2022 using a syndromic surveillance network of GPs. We observed the seasonality of HFMD incidence during the non-COVID years, with a peak of mean weekly rates between September and December. Our data shows that in England, children aged 1–4 years old accounted for the largest affected population followed by children <1 years old. This study shows that syndromic surveillance GP reporting on a near-real-time basis can provide valuable insight into HFMD epidemiology. The experiences and lessons learnt from other countries where large outbreaks have occurred (including virulent strains and therefore more severe presentations and increased mortality) highlight the importance of understanding the evolving aetiology of HFMD, epidemiology and changing burden of clinical cases. We have shown that monitoring changes in HFMD epidemiology through prospective surveillance can also provide timely alerts in the event of increasing activity both at the national, regional or local levels that might implicate changes in the underlying aetiology of cases. Our data provides the framework for assessing changes in healthcare presentation linked to future changes in the presenting severity of cases.

## Data Availability

Applications for requests to access relevant anonymised data included in this study should be submitted to the UKHSA Office for Data Release (https://www.gov.uk/government/publications/accessing-ukhsa-protected-data/accessing-ukhsa-protected-data).
